# Molecular Aspects of an Emerging Poplar Canker Caused by *Lonsdalea populi*

**DOI:** 10.3389/fmicb.2019.02496

**Published:** 2019-11-06

**Authors:** Aining Li, Wei He

**Affiliations:** Beijing Key Laboratory for Forest Pest Control, College of Forestry, Beijing Forestry University, Beijing, China

**Keywords:** poplar canker, *Lonsdalea populi*, virulence, virulence factors, poplar-bacterium interaction

## Abstract

The Gram-negative bacterium *Lonsdalea populi* causes a lethal disease known as bark canker on *Populus* × *euramericana* in China and Europe. Typical symptoms of bark canker include an abundant white-colored fluid, which oozes from the infected tissues. The availability of the genomic sequence of the bacterium provided the necessary resource to launch genome-scale investigations into the mechanisms fundamental to pathogenesis. Functional analyses of a diverse group of genes encoding virulence factors and components of signaling pathways indicate that successful bark infection depends on specific responses by the pathogen to various stresses, including oxidative stress. Although physiology of resistance is well studied, the molecular processes underlying the defense responses and the genetic basis of resistance to *L. populi* and in other poplar species remain largely unknown. Control of the disease has relied on chemical measures. Due to the genetic amenability of *Lonsdalea* and poplar, this pathosystem will become an important model system to unravel molecular mechanisms of bacterial pathogenicity on woody plants. Increased understanding of pathogenesis and signaling in the interaction will facilitate the management of this kind of poplar canker.

## Introduction

Poplars are predominantly distributed throughout the northern hemisphere. Because of their rapid growth, wild *Populus* spp. and their hybrids are currently planted over huge areas worldwide as ornamental plants for landscape greening, production of wood, and multiple industrial uses (Stettler et al., 1996; Jansson and Douglas, 2007). *Populus* × *euramericana*, an interspecific hybrid poplar, is one of the most widely grown poplars in China. However, poplars are often attacked by insects and various microbial pathogens. Among them, cankers represent the most destructive poplar diseases, which are localized in the bark of poplar trunk and branches. *Cytospora chrysosperma* and *Botryosphaeria dothidea* are the main fungal pathogen of poplar cankers worldwide (Sinclair and Lyon, 2005; Fan et al., 2019). In the 1990s, the bacterial genus *Brenneria* was also noted to cause canker of trees in Spain, including poplar (Biosca et al., 2006). Currently, a large portion of the *P*. × *euramericana* plantation area in China and Hungary is affected by one potentially lethal bacterium, *Lonsdalea populi* (formerly *Lonsdalea quercina* subsp. *populi*; Toth et al., 2013; Li et al., 2014).

**Figure 1:**
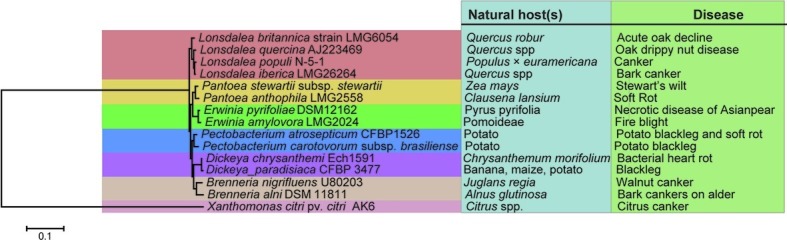
Phylogenetic tree based on 16S ribosomal RNA gene sequences of *Lonsdalea* species and their closest phylogenetic neighbors. Sequences of 16 s rDNA of *Lonsdalea populi* N-5-1 (JF424600), *Lonsdalea iberica* (JF311441), *Lonsdalea quercina* (AJ223469), *Lonsdalea britannica* (JF311446), *Brenneria alni* (AJ233409), *Brenneria nigrifluens* (U80203), *Erwinia amylovora* (Z96088), *Erwinia pyrifoliae* (AJ009930), *Dickeya paradisiaca* (AF520710), *Dickeya chrysanthemi* (NR_117738), *Pantoea anthophila* (EF688010), *Pantoea stewartii* subsp*. stewartii* (KM508089), *Pectobacterium carotovorum* subsp*. brasiliense* (NR_118228), *Pectobacterium atrosepticum* (NR_118295), and *Xanthomonas citri* pv*. citri* (LC202834). The scale bar indicates a 0.1 nucleotide changes per site. *X. citri* pv*. citri* was included as outgroups. The principal host plants and diseases were shown next to the tree branches.

This causal agent of poplar canker was first detected by Toth et al. (2013) in Hungary and later described by Li et al. (2014) in China as *Lonsdalea populi,* formerly known as *Lonsdalea quercina* subsp. *populi* (Li et al., 2017). The bacterium belongs to the genus *Lonsdalea*, which is a novel genus following the removal of the species *Brenneria quercina* from this classification (Brady et al., 2012). Furthermore, *B. quercina* was renamed from *Erwinia quercina*, which caused drippy nut disease of native live oaks in California (Hauben et al., 1998). There are distinctive biochemical and physiological characteristics among the genera of *Lonsdalea*, *Brenneria,* and *Erwinia*, which distinguish each from one another (Toth et al., 2013). Until recently, *Lonsdalea quercina* contains four subspecies (*L. quercina* subsp. *populi*, *L. quercina* subsp*. quercina*, *L*. *quercina* subsp*. iberica*, and *L. quercina* subsp*. britannica*), which are all pathogens of woody trees (Toth et al., 2013). Due to the genome sequence-derived average nucleotide identity (ANI) values between the four subspecies were 88.71–93.38%, respectively, lower than the proposed species boundary ANI cut-off (95–96%) that is considered the most important criterion to reclassify these subspecies at the species level. It is proposed that three subspecies were elevated to the species level, among them *L. quercina* subsp. *populi* was substituted to *L. populi* sp. nov. (Li et al., 2017). Using 16 s rDNA analysis, we reconstructed the phylogenetic tree (Figure 1) containing those sequences from four *Lonsdalea* species and other related sequences. This tree also suggests the close relationship of *Lonsdalea* species to *Erwinia amylovora* and *Pantoea agglomerans*, two major phytopathogenic bacteria.

In 2006, severe bark canker symptom on *P*. × *euramericana* was observed for the first time in Henan and Shandong provinces of China. In 2009, the same symptom was also detected in *P.* ×*euramericana* stands in the central part of Hungary (Toth et al., 2013). Currently, a large part of the *P*. × *euramericana* plantation, including in Ningxia and Tianjin of China, is affected by this disease. Although a preliminary report showed that the fungus *Fusarium solani* was identified as a pathogen of this canker disease in 2009 (He et al., 2009), it is now beyond dispute that *L. populi* is the causal agent of *P*. × *euramericana* canker in China and Europe (Li et al., 2017). The availability of the genome sequence and genetic manipulation platform has accelerated rapid advances into functional genomics of pathogenicity and stress responses. Both the poplar host and the bacterium serve as excellent models for studying the molecular basis of poplar and woody tree-bacterial interactions.

The main aim of this review is to summarize the current state of knowledge concerning the emerging poplar canker. The review provides information on the following topics: (1) disease symptoms and host range; (2) techniques for pathogen detection; (3) functional characterization of virulence factors such as type III secretion and two-component systems; (4) disease management; and (5) perspectives and implications from other pathosystems. This review incorporates what is currently known on the molecular aspects of the poplar-bacterium interaction.

## Symptoms of *Lonsdalea* Canker and Host Range

**Figure 2:**
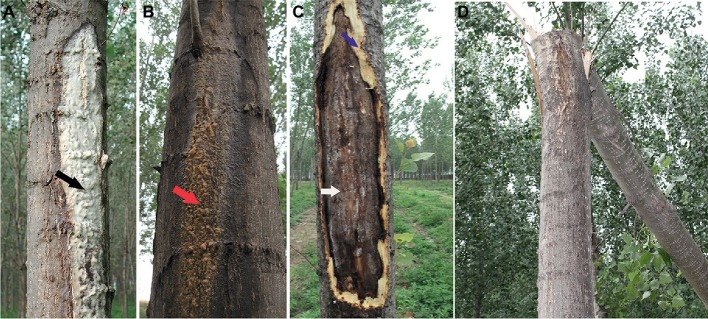
Symptoms and damage of canker disease of *Populus* × *euramericana* caused by the bacterial pathogen, *Lonsdalea populi*. **(A)** Early symptoms with abundant sour and white, sour exudate (black arrow) on poplar trunk; **(B)** Late brown bark symptoms in the summer month, brownish exudations (red arrow). **(C)** Overwintering canker with large bark necrosis (white arrow) and fresh necrotic bark (blue arrow). **(D)** Diseased poplar blown down by the wind in the field.

Poplar canker caused by *L. populi* is essentially lethal disease, affecting stems or branches of *P*. × *euramericana* that are typically more than 3-year olds in the field (Figure 2). Symptom development on *P*. × *euramericana* “74/76” starts in early summer with the appearance of abundant, white, sour exudates on the stem (Figure 2A), which is associated with lesions. The lesions expand vertically, causing cracking and also forming a sticky brown-colored fluid oozes with the rotten smell (Figure 2B). Most diseased poplars show a cracked stem or branch, exuding frothy fluid (Figure 2C; Li et al., 2014). The cankers expand rapidly, and in serious cases, poplars with these cankers may die, or entire branches may be broken off by wind (Figure 2D).

The incubation period until the appearance of symptoms, following bacterial inoculation of excised stems of poplar, lasts about 3 days, although water-cultured excised stems show initial symptoms after 4 days (Shang et al., 2014). As soon as the whitish-yellow-colored liquid flowed from the inoculation site, the necrotic lesion preferentially expanded longitudinally. The infection spreads within the central tissues including *via* xylem, tissue which is frequently associated with a brownish discoloration. *L. populi* was detected by PCR amplification with species-specific primers ahead of the visible necrotic lesion in apparently healthy inner bark tissues and reached peak quantities, however, when associated with the severe necrotic symptom (Shang et al., 2015). Real-time PCR analysis showed that *L. populi* mainly exists in the phloem tissue, as well as exudates of canker, but not in xylem tissue or the leaves, and the peak bacterial accumulation occurs at 7 days after infection when the phloem tissue was seriously damaged and parenchyma cells were disaggregated (Shang et al., 2014). In field conditions, lesions on the bark associated with this disease mostly become visible during late spring and summer. The beetles, *Librodor japonicus* and *Protaetia brevitarsis*, were found with the ooze associated with the cankers. Furthermore, DNA of *L. populi* can be detected in the beetles from infected trees but not uninfected trees by PCR amplification, thus suggesting that these insects could be helpful in spreading the disease to other trees.

*Lonsdalea* canker was initially found on four cultivars of *P*. × *euramericana* in China, such as “Zhonglin 46,” “74/76,” “Zhonghe 1,” and “Robusta.” Among them, “Zhonglin 46” is the most susceptible, and the incidence on this cultivar can rise up to 70% (Li et al., 2014). Although *L. populi* is primarily restricted to *P*. × *euramericana*, inoculation with the bacterial inocula in the lab leads to bark canker of a wide range of poplar cultivars, including *Populus tomentosa*. In 2017, *Lonsdalea* poplar canker was recorded in Portugal (Abelleira et al., 2019). Moreover, *Populus* × *interamericana* “Beaupre” and *P.* × *euramericana* “I-214” and “MC” were infected by *L. populi* in nine plantations in Spain (Berruete et al., 2016). Recently, willow trees (*Salix matsudana*) infected naturally, and stem inoculation experiments using healthy willow trees showed that *S. matsudana* was also susceptible to *L. populi* (Li et al., 2019).

## Techniques for Pathogen Detection

Isolation of *L. populi* from necrotic lesions on the bark is straightforward, although *F. solani* has sometimes been found (He et al., 2009; Li et al., 2014). A tiny tissue sample is aseptically taken from the xylem close to the necrotic bark after the samples were shaken in sterile distilled water for 10 min and incubated on Luria-Bertani medium. Bacterial isolation success largely depends on the season and the age of the canker lesion. Fresh canker usually appears in the late spring and summer, and isolation yields good success. During the winter months, the ratio of bacterial isolation remains lower.

16S rRNA and three housekeeping genes (*gyrB*, *atpD*, and *infB*) can be used as target sequences to differentiate the bacterium from the close species, including *L. iberica* and *L. britannica* (Toth et al., 2013; Li et al., 2014). Based on comparative genomic sequence analyses, two species-specific primers (LqfF/LqfR and LqgF/LqgR) are available for the rapid and accurate detection of the bacterium in diseased plant tissues, and a real-time PCR assay for *in planta* detection at the minimum amount of 0.1 pg. genomic DNA of *L. populi* has been developed (Shang et al., 2015). These methods were developed to monitor asymptomatic poplar before typical symptoms appeared.

## Functional Characterization of Virulence Genes Associated With the Type III Secretion and Two-Component Systems

Research into the molecular biology of virulence has been spurred forward by the availability of genomic resources and the ease of genetic transformation to obtain mutants of *L. populi*. The genome size of *L. populi* N-5-1 is 3.9 Mb with G + C content of 55.42% and contains 3,746 predicted protein-encoding genes (Table 1). The bacterium is highly amendable to molecular genetic manipulation, especially insertional inactivation and in-frame deletion methods. Below, we provide and discuss an overview of the current knowledge of *L. populi* pathogenesis from a genomic perspective.

**Table 1:** Summary of *Lonsdalea populi* N-5-1 genome sequencing.

Genome size (bp)	3,905,180
GC content (%)	55.42
Number of predicted genes	3,746
Gene total length	3,155,847
Repetitive sequence (bp)	4,691
tRNA	67 (5,266)
5 s rRNA	6 (690)
16 s rRNA	2 (3,062)
23 s rRNA	3 (8,715)
CAZy	88
Secreted proteins	225

**Figure 3:**
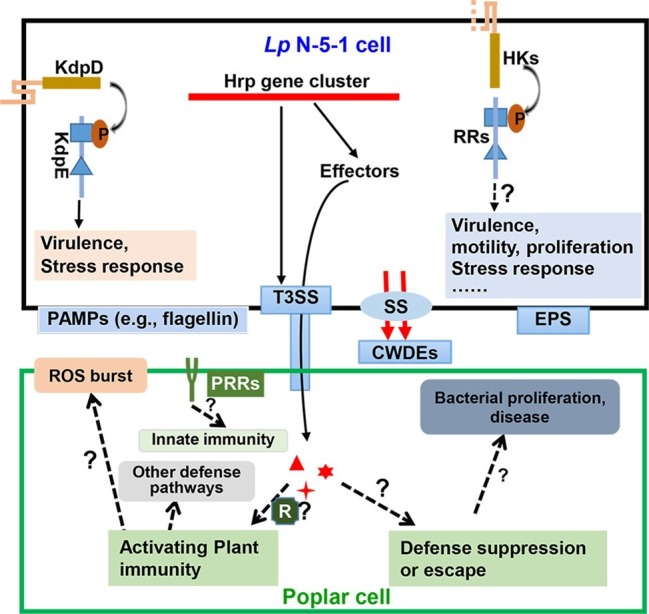
A diagram of the two-component signaling and secretion systems during interaction of *Lonsdalea populi* and *Populus* × *euramericana*. The bacterium *L. populi* N-5-1 mainly perceives external signals *via* two-component systems (TCS) comprising histine kinases (HKs) and response regulators (RRs). This leads to activation of TCS signaling that modulates expression of genes associated with virulence, motility, proliferation, stress response, etc. A well-characterized TCS of *L. populi* N-5-1, KdpD-KdpE, has been demonstrated to control virulence and stress response (Yang et al., 2018). Moreover, *L. populi* N-5-1 contains a 23 kb gene cluster encoding a type III secretion system (T3SS), which is deployed along with other secretion systems (SS) to deliver effectors and plant cell wall-degrading enzymes (CWDEs), which may activate poplar plant immunity. In response, the host may release a reactive oxygen species (ROS) burst and other defense pathways, or some effectors in susceptible hosts may promote bacterial infection and cause canker disease. Exopolysaccharides (EPS) also facilitate bacterial virulence. On the other hand, plant pattern recognition receptor (PRR) complexes recognize pathogen-associated molecular patterns (PAMPs), which result in activation of plant innate immunity. In this view, an undetermined number of transmembrane domains of HK is shown by dashed lines. Dashed arrows and lines indicate potential regulation. Question marks indicate predicted characteristics that are yet to be substantiated.

The type III secretion system (T3SS) comprises major virulence factors in many Gram-negative bacterial pathogens and is usually encoded by a gene cluster associated with pathogenicity islands (Hacker et al., 1997). Bacteria deploy T3SS to deliver various effectors into the host cell, which can manipulate various plant processes to promote infection or activate plant immunity system. Genome analyses of the strain N-5-1 revealed a 23 kb T3SS gene cluster. The genetic organization of this cluster is highly similar to that of the cognate gene clusters in *Dickeya dadantii* 3,937 and *Erwinia amylovora* CFBP1430, whereas it is strikingly different from that observed in *Ralstonia solanacearum* GMI100 (Yang et al., 2014b). This cluster contains 26 *Hrp* genes related to the hypersensitive response on non-hosts and that contributes to pathogenicity. Nine of these genes encode highly conserved Hrc proteins, while others act as effectors and regulators of T3SS. *HrcW* mutants exhibited reduced virulence on poplar and heterogeneous expression of N-terminal but not C-terminal HrpW triggered hypersensitive response (HR) on tobacco leaves (Yang et al., 2014a). In addition, *HrcV* encoding a T3SS structural protein was required for virulence on poplar stem and HR on tobacco leaves, while HrcV played no obvious roles in bacterial growth, motility, and biofilm formation (Yang et al., 2014b). A third Hrp gene, *HrcJ*, was characterized by insertional mutagenesis and was required for pathogenicity of *L. populi* on poplar and to elicit an HR response in tobacco. The *HrcJ* deletion mutant did not affect growth and biofilm formation but significantly decreased the motility (Li et al., 2015). These data document the importance of T3SS in pathogenesis, but it remains to be shown how T3SS is connected to virulence and xylem colonization of poplar plants in *L. populi*, as shown in Figure 3.

In bacteria, two-component signal transduction systems (TCSs) play essential roles in the regulation of physiological and cellular processes, including virulence and adaptation (Stock et al., 2000). A typical TCS comprises a membrane-bound histidine kinase (HK) and a cytosolic response regulator (RR) (Capra and Laub, 2012). BLASTp searches were undertaken to determine how many genes are involved in conserved HK-RR phosphorylation, uncovering 18 HKs and 24 RRs (Yang et al., 2018). Interestingly, TCS genes are thought to account for approximately 2 or 3% of the annotated genes of a bacterial genome (Wuichet et al., 2010). Compared with the number of TCSs with other bacteria of the Pectobacteriaceae family, the genome of *L. populi* encodes a relatively smaller number of TCSs. Further analyses of TCSs with the set of 18 HKs and 24 RRs revealed 15 pairs of HK and RR genes that were located close to each other in the genome and 12 orphan TCS genes (3 HKs and 9 RRs), which were not clustered with any of the other HK and RR genes in the genome of *L. populi*. A large-scale insertional mutagenesis study resulted in 32 mutants (16 HK and 16 RR mutants) and further demonstrated that 19 TCS genes regulated bacterial virulence against poplar trees. Among these, a novel TCS, KdpD-KdpE, formed a bi-cistronic operon, which not only controls bacterial virulence, growth, and swimming motility but also regulates bacterial resistance to oxidative and chloramphenicol stresses (Yang et al., 2018). Furthermore, KdpE functions as a transcription factor, which binds to the promoters of 44 genes, including stress response genes. Recently, an orphan RR, LqRR2, has been further characterized, revealing that it is required for virulence and bacterial motility, possibly regulating the expression of *flgB*, *flgC,* and *flgE*, rather than being directly involved in the biofilm formation and extracellular polysaccharide production (Li et al., 2018). Consequently, *L. populi* TCSs, more HK-RR-like proteins, and KdpD-KdpE or orphan proteins require identification and characterization and also further analyzes the environmental or host stimuli that are recognized and transduced by TCSs. Biochemical and structural investigation could be applied to comprehensively characterize the molecular mechanisms orchestrated by TCSs.

The recent studies into the molecular aspects of virulence in *L. populi* have addressed a range of questions from T3SS effectors to TCS and stress responses. Future research into the functional genomics of pathogenicity and host adaptation will include a better understanding of molecular bases underlying multiple secretion pathways, secondary metabolite production, biofilm formation, and manipulation of host susceptibility, as summarized in Figure 3.

## Poplar-Bacterium Interaction

To better characterize the interaction between *L. populi* and poplar plants, physiological and molecular responses in that plant have been assessed. Initially, the expression of *PR1*-1, *PR1*-2, *NPR1*-1, *NPR1*-2, *TGA1*, *TGA2*, *MYC2*-1, and *MYC2*-2 was recorded as higher in a resistant poplar variety (*Populus tomentosa*) than the susceptible one (*P. deltoids* cv. “Zhonghe 1”), while *JAZ1*, *COI1*-1, and *COI1*-2 were downregulated in the resistant variety, suggesting the involvement of SA and JA signal transduction during the infection of the bacterium (Liu et al., 2015). Whether the SA and JA pathways are necessary or sufficient for resistance remains to be determined, the transcriptome analysis is clearly consistent with their involvement. The transcriptomic profiles of poplar in response to infection revealed a potential role of the biosynthesis of secondary metabolites, plant hormone signal transduction, regulation of autophagy, and ABC transporters during *L. quercina* infection (Hou et al., 2016). In addition, the study also demonstrated that flavonoid biosynthesis pathway and plant hormone signal transduction might play important roles in poplar response to the infection (Hou et al., 2016). Recently, RNA-Seq analysis at the early stage of poplar response upon *L. quercina* infection showed that Vitamin B6 metabolism, selenocompound metabolism, and benzoxazinoid biosynthesis pathways of poplar were induced and also indicated that flavonoid and phenylpropanoid would be involved in the poplar defense response to the infection at the early stage (Zhou et al., 2019). While the transcriptomic analysis of the poplar response to bacterial infection has been characterized in some detail, significantly less is known regarding the response of the bacterium during infection and colonization of poplar. Dual-RNA sequencing of host and pathogen will provide novel insights into the interaction between poplar and bacterium. Moreover, a striking gap of knowledge on the interaction between poplar R genes and bacterial effectors. Although hundreds of R genes of poplar and a dozen of putative T3SS effectors in the pathogen have been identified, what are the molecular bases of the interaction and how does the interaction determine poplar resistance or susceptibility (Figure 3)?

## Disease Management

Integrated disease management strategies for *Lonsdalea* canker need to minimize poplar losses without raising the specter of environmental concerns. Due to the limited host range of *Lonsdalea* canker, breeding resistant poplar varieties or deployment of resistant species such as *P. tomentosa* would be a preferred solution to control the disease. At present, little is known about R-mediated resistance against *Lonsdalea* canker, although resistant poplar does exist in the field. Until now, *P. tomentosa* is the most resistant, and 74/76 is medium resistant, while “313,” “Tianyan,” and “599” are the most susceptible. Other control measures have also been explored. For example, Xu reported a potential approach of biological control to use *Bacillus subtilis* P2 or *Streptomyces venezuelae* P4, which was isolated from rhizosphere soil of *Lonsdalea* canker of poplar (Xu et al., 2013). Typically, however, practical implementation of biocontrol organisms under field conditions remains a bottleneck. Some antibiotics, such as polyoxin, streptomycin, ethylicin, and amobam, were screened and exhibited obvious effectiveness to inhibit the pathogen and canker expansion in the lab and under field conditions (Xu et al., 2013). Meanwhile, the application of antibiotics to control this disease on a large scale and the potential for the development of antibiotic resistance in the pathogen should be carefully considered.

## Implication From Other Pathosystems and Perspectives

Although the underlying mechanisms of the poplar – *L. populi* pathosystem appear unexplored, comparisons with other bacterial pathosystems might provide a useful reference of the understanding and control of *Lonsdalea* canker. Citrus canker disease is caused by *Xanthomonas* strains (Brunings and Gabriel, 2003), and the disease that these strains cause is one of the most economically damaging diseases affecting citrus products worldwide, and the presence of citrus canker in an area triggers immediate quarantine restrictions (Graham et al., 2004). Integrated approaches for prevention and control of citrus canker have been used, but outbreaks of the disease continue to occur (Graham et al., 2004). Fire blight disease, caused by *E. amylovora* disease, is incredibly destructive to the fruit industry worldwide (Eastgate, 2000). A lesson from both tree bacterial diseases is the importance of avoiding further introductions of pathogenic strains, the significance of discovering pathogenicity determinants of pathogens, and the major resistance genes of hosts. Therefore, even if *L. populi* has only been discovered in relatively small regions across the globe, prohibiting the importation of plant material from canker-endemic areas is advised. In addition, it is important to study the genetics, physiology, and molecular mechanisms of the pathogen and poplar, especially in how the molecular interactions between the two can affect disease outcomes.

Although this review has revealed considerable advances in our understanding of the molecular basis of *L. populi* virulence and stress response, many questions remain unsolved. The molecular events underlying the infection process and overwintering in cankers have caught little consideration. On the one hand, many molecular tools of the bacterium have been established. On the other hand, poplar is an excellent model for trees. Therefore, the development of more effective controls for this devastating disease is a worthy goal for the future, and the poplar – *L. populi* pathosystem might become a model of forest pathology.

## Author Contributions

AL designed the experiments. AL and WH analyzed data and wrote the manuscript.

### Conflict of Interest

The authors declare that the research was conducted in the absence of any commercial or financial relationships that could be construed as a potential conflict of interest.
